# Self-Propelled Vehicles

**Published:** 1907-02

**Authors:** 


### SELF-PROPELLED VEHICLES.

A practical treatise on all forms of automobiles, by **James E. Homans, A. M., Fifth Revised Edition, entirely rewritten. New York, Theo Audel & Co., 63 Fifth Avenue, 1907.**

New and complete revised, this popular book fulfils the requirements of the motor vehicle owner, operator and repairer.

In his revision, the author has emphasized the practical aspects of motor vehicles of all powers, confining his space to the discussion of matters fundamental in construction and management.

Theoretical matters—important almost wholly to designers and builders—are introduced only where good explanations positively required them, and at no point is the reader's mind burdened with padded material on experimental and obsolete construction.

Recognizing that the gasoline vehicle is the typical automobile, considerable space is devoted to its complete discussion; theory, operation, and an extensive chapter on "Gasoline Engine Management," the latter covering, virtually, all forms of difficulty liable to occur under service conditions. Anyone reading this chapter can derive an intelligent conception of the requirements for an expert driver, and will find numerous points of information, usually obtainable only by long and varied experience.

All the accessory parts of an automobile, carburetters, igniters, transmission gears, are fully explained by typical examples. The author properly assumes that an adequate knowledge of the principles upon which these devises are construceted will nabl the reader to understand variations for himself.

To sum up the excellencies of this book, it must be acknowledged that it fulfils the ideal of the practical motor driver's vade mecum. All subjects are fully illustrated; as to its standard of book making, it may be said to be almost unequaled in paper, type-work and binding.

It is admirably suited as an educational treatise and will doubtless be widely used in that field.

---

John Rose Bradford, M. D., F. R. C. P., in dealing with Diseases of the Kidneys, says: "Functional albuminuria is a condition of great importance to all practitioners, both from a point of view of diagnosis and of treatment. Certainly, it would seem at the present time that many of these cases are submitted to far more rigorous treatment, especially in diet, than is necessary." Paroxysmal Hemoglobinuria, the Retention of Chlorides in Nephritis, and more particularly the treatment of Nephritis are carefully considered. This section concludes with an article on renal dropsy.

Dr. Joseph C. Bloodgood devotes one hundred pages to Fractures, Dislocations, Amputations, and the Surgery of the Extremities. The healing of fractures, the problems presented by fractures at the lower end of the humerus, metacarpal fractures and sprains of the ankle-joint are all thoroughly discussed. So with Osteomyelitis, Paget's disease, and bone tumors. Bier's Treatment with Hyperemia is detailed at some length, and this section is freely illustrated. His conclusions in regard to the treatment of Tuberculosis of the bones and joints are: "I believe, if these patients come to treatment in the early stage, that open air combined with Bier's hyperemia and proper orthopedic apparatus will accomplish an ultimate cure in an increasing proportion of cases, and operations will be performed, relatively, less frequently." Dr. H. R. M. Landis contributes over sixty pages to the Therapeutic Referendum. Every page is bristling with therapeutic facts of practical importance. "It has now been established beyond a reasonable doubt that in tetanus antitoxin we possess a certain means of preventing tetanus, and that this serum should be used after all wounds in which there is any possibility to tetanus developing." Many useful suggestions are made in regard to drugs. Cases of poisoning by so-called "headache powders" are cited and the possibly injurious effects of adrenalin if used continuously. So through alcohol, ergot, iodine, mercury, mushroom poisoning, nitre, opium, the salicylates, urotropin and many other drugs, the interest is maintained while many important facts are logiclly developed.

---

*THE ORIGIN OF SLEEP.*—By Dr. Salmon (Revue De Medicine, No. 4, 1906).

The author studied the relations existing between sleep and the function of the pituitary gland. He believes the function of sleep to be an internal physiological secretion. The hypophysis is assumed to play an essential part in the production of sleep. Since the substance of this gland is said to contain bromin, this serves to support the theory that the hypophysis exerts a sleep-inducing influence upon the nerve centers. The author endeavors to back up his statements by the occurrence of anatomical changes in this organ in various diseases, which are associated either with somnolence or with insomnia.

